# Paragraphs

**Published:** 1882-12

**Authors:** 


A New Floor Covering.—A new and desirable papier maché process for covering floors is described as follows : The floor is thoroughly cleaned. The holes and cracks are then filled with paper putty, made by soaking newspapers in a paste made of wheat floor, water and ground alum, as follows: To one pound of flour add three quarts of water and a tablespoonful of ground alum, and mix thoroughly. The floor is then coated with this paste, and then a thickness of manilla or hardware paper is put on. If two layers are desired, a second covering of manilla paper is put on. This is allowed to dry thoroughly. The manilla paper is then covered with paste, and a layer of wall paper of any style or design desired is put on. After allowing this to thoroughly dry it is covered with two or more coats of sizing, made by dissolving one-half pound of white glue in two quarts of hot water. After allowing this to dry, the surface is given one coat of "hard oil-finish varnish," which comes and is bought already prepared. This is allowed to dry thoroughly, when the floor is ready for use. The process is represented to be durable and cheap, and besides taking the place of matting, carpet, oil cloths or like covering, makes the floor air tight, and can be washed or scrubbed.

Potato Bugs.—Parties who are fighting potato bugs will be glad to read and then try the following remedy, which the *Troy Press* says has been found unfailing : He procured a number of boards, and placed them here and there among his potatoes, and on the boards were placed raw potatoes sliced. At noon the first day of the experiment he and his hired men found every piece of potato covered with bugs. The men killed this crop, and at night another crop was killed, though not so large, and in a week's time not a bug could be seen, and his troubles with bugs after this was comparatively small. In the spring, he says, is the best time to attend to the bugs, as spring bugs, he understands, breed from three to five hundred during the potato season. He thinks it would be a good plan to dip the pieces of potato in Paris green, as it would save the work of killing the bugs.

Coal Product of Illinois.—The State Bureau of Labor shows that Illinois ranks next to Pennsylvania in the production of coal. The output for 1882 was 9,000,000 tons. Last year the yield was 6,000,000 tons. The coal mines are found in forty-six of the hundred counties of the State. The value of the year's yield of coal at the mines was nearly $14,000,000.

Simple Fire Escape.—A well-known army officer, who travels a great deal in the West, has a part of his trunk arranged to hold a knotted rope. When he stops at a hotel, he takes out the rope, fastens one end to the bedstead, or some heavy piece of furniture, and puts the rest of the rope in a pail of water placed directly under the window, so that it may be quickly thrown out, the object of wetting the rope being to prevent its being burnt off readily by flames from a window below.

A Southern Cure for Sunstroke.—So soon as you reach your patient take hold of him or her and carry or drag him or her into the shade. Place the body in a sitting posture, the back against a wall, with the feet and legs resting upon the sidewalk and extending in front of the body. Get ice-water and a bottle of some strong essence of ginger. Pour the ice-water over the head, copiously; never mind the clothes. Then pour two or three tablespoonfuls of ginger in about half a tumbler of water and make the patient swallow it quickly. Keep the head cool by using a little of the ice-water, and in case there is not much of a glow upon the body give more ginger. If this recipe is promptly used and fully carried out in every case, the Board of Health will never have a death to record from this cause. It is no experiment or quack remedy. It costs but a few cents and a half hour or an hour's time. Ginger is by far the best to use, but where it cannot be had quickly two or three good drinks of brandy will answer.—*N. O. Picayune.*

